# HTML5 PivotViewer: high-throughput visualization and querying of image data on the web

**DOI:** 10.1093/bioinformatics/btu349

**Published:** 2014-05-21

**Authors:** Stephen Taylor, Roger Noble

**Affiliations:** ^1^Computational Biology Research Group, Weatherall Institute of Molecular Medicine, University of Oxford, John Radcliffe Hospital, Headington, Oxford OX3 9DS, UK and ^2^Coritsu Group, Adelaide, South Australia 5000, Australia

## Abstract

**Motivation:** Visualization and analysis of large numbers of biological images has generated a bottle neck in research. We present HTML5 PivotViewer, a novel, open source, platform-independent viewer making use of the latest web technologies that allows seamless access to images and associated metadata for each image. This provides a powerful method to allow end users to mine their data.

**Availability and implementation:** Documentation, examples and links to the software are available from http://www.cbrg.ox.ac.uk/data/pivotviewer/. The software is licensed under GPLv2.

**Contact:**  stephen.taylor@imm.ox.ac.uk and roger@coritsu.com

## 1 INTRODUCTION

Bioimaging and associated informatics are generating unprecedented amounts of data. New sophisticated imaging techniques yield large, heterogeneous, multidimensional datasets that need to be viewed, analysed, annotated, queried and shared (Carpenter *et al.*, 2012).

Development of automated image analysis methods has been facilitated by various tools such as ImageJ/Fiji (Girish and Vijayalakshmi, 2004) using the built-in macro functions, but when there are large amounts of data and associated images, reviewing and analysing the results is burdensome. There are no powerful and intuitive web tools available that allow filtering and sorting of images based on the derived properties of these images. OMERO (Allan *et al.*, 2012) provides a comprehensive database for storing and viewing large amounts of images, however, viewing and querying thousands of images using the OMERO.insight client or OMERO.web is slow.

Microsoft Live Labs Pivot has been used in a neuroimaging setting (Viangteeravat *et al.*, 2011) but the Silverlight version, although powerful, required a specific plug-in and was not accessible on all platforms and cannot be easily extended. In addition, support for Silverlight is being phased out.

Our goal was to develop an open-source extensible viewer specifically designed using the standard HTML5 canvas element and JavaScript technologies. This enables informaticians to create dynamic and interactive visualizations of the results of image analysis or large image datasets, providing a powerful but simple and intuitive front end that works in any modern web browser. It allows users to see their data, filter, sort and identify relationships based on the metadata supplied for each image. Because the technology is based on open standards, there is potential to integrate with other HTML5-based libraries, such as D3 (http://d3js.org/), iCanplot (Sinha and Armstrong, 2012) for statistical visualization and Scribl (Miller *et al.*, 2013) for multiple region genomic visualization.

## 2 IMPLEMENTATION

### 2.1 Background

To set up a HTML5 PivotViewer instance, a directory of JPG or PNG images is required, each with unique ids. A separate tab-delimited file containing the unique id of the image, path to each image and a series of columns containing properties or ‘facets’ of each image is used as input to the indexing software.

Perl and Python indexing scripts are provided that allow generation of metadata XML files for the query engine and a series of XML files that specify image properties such as tile sizes, zoom levels and pointers to the images in the tiling pyramid. The tiling pyramid generated by the scripts is a series of images where each layer in the pyramid corresponds to a zoom level split up into various subimages. The maximum level for an image is determined by *log_2_ max*(*width, height*).

HTML5 PivotViewer only loads the appropriate subimage when required, making it well-suited for displaying high-resolution images in low-bandwidth and mobile applications.

### 2.2 Architecture

HTML5 PivotViewer is a plug-in to the jQuery JavaScript library requiring a parent element which the control can be attached to. The control has been built with extensibility in mind and has been constructed to allow for various types of data sources, facet types and visualizations. The core visual elements of the control have been built around the HTML5 canvas element, which provides a surface for raster-based graphics and pixel-level image manipulation, which can render at flexible frame rates determined by the parent browser and platform.

### 2.3 Extensibility

There are three areas of functionality that can be enhanced: Loaders, Facet Types and Views. Loaders are used to load data from various sources and transform them into the HTML5 PivotViewer's internal data structures. The control has implementations for CXML (Collection XML for collections structured by the http://schemas.microsoft.com/collection/metadata/2009 schema). Additional loaders could include support for CSV, TSV or web services. Facet types describe the data types contained within an item and have a 0-to-many relationship with their parent. Current facet types include String, Numeric, DateTime and URIs. Views represent the application logic used to render the collection items to the HTML5 canvas element. The default grid and histogram view allow for sorting and organizing the items into buckets. Additional data visualizations could be developed; such as scatterplots, treemaps, dendrograms or heatmaps.

### 2.4 Example applications

**Fig. 1.**
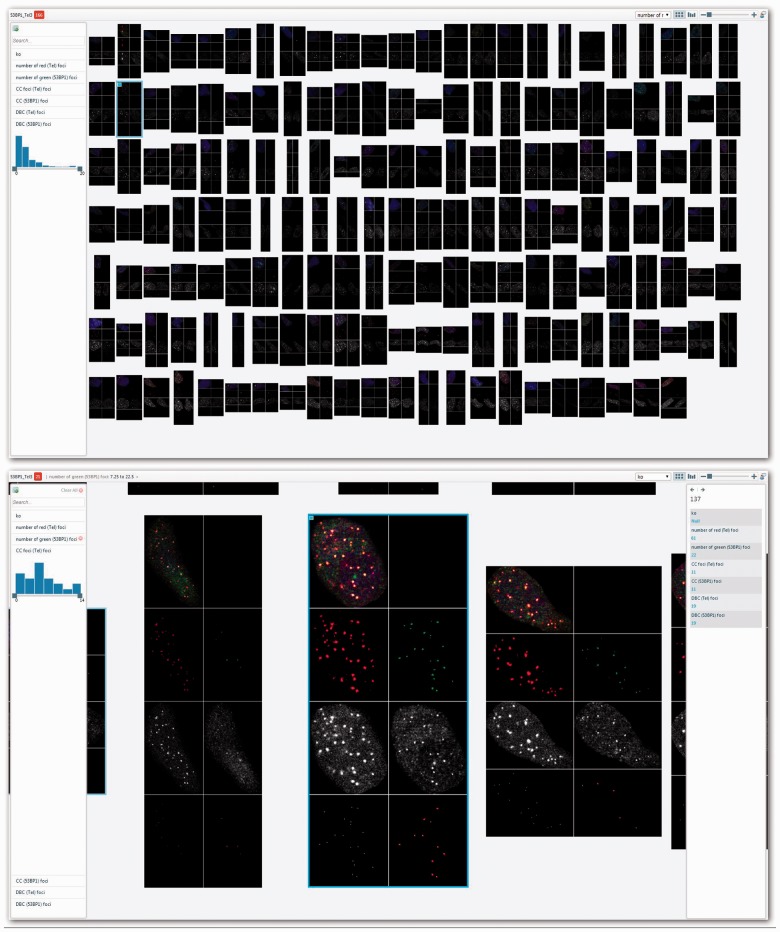
HTML5 PivotViewer display. The upper image shows 166 montages derived from colocalization analysis. The query controls are visible on the left panel. The lower image shows the zoomed-in view with associated metadata in the right panel. Images can be sorted in the viewer using any associated metadata

Any set of images that have associated metadata, such as segmented cells, wells, tissue or organ samples, are amenable to be used in HTML5 PivotViewer. Figure 1 shows a colocalization analysis example.

In this dataset (Clynes *et al.*, 2014), ImageJ macros were developed that processed raw wide-field microscope images, segmenting DAPI (blue) stained nuclei into individual multichannel images. We retain all the original image files for reference and use a simple directory structure to organize the data. The cells were labeled with florescent antibody (red and green) relating to the proteins that were being investigated. Each segmented image was auto-thresholded in the red and green channel and then analysed for colocalization using the JaCoP (Bolte and Cordelieres, 2006) plug-in. The final output was a PNG montage of each nuclei showing the raw, red and green channels, and JaCoP output showing coincidental foci and overlapping foci for each antibody. In a typical batch, there were ∼100–200 images and the user could rapidly check the automated algorithms have counted the foci accurately, graph the numbers of red or green foci, filter colocalizing foci above a certain threshold and export the filtered results to a tab-delimited text file for further analysis. Other example applications are available on the supplied website link.

## 3 DISCUSSION

HTML5 PivotViewer has many applications in biology and any discipline that requires the ability to drill down through many images in the context of their metadata. It can comfortably handle at least 1000 images, providing informaticians a platform to share results of complex analyses on the web and end users a simple and compelling way to manage, explore and understand large image-based datasets. Future work will include handling increased numbers of images and being able to import and view other image types (such as stack and movie formats) within the software.

## References

[btu349-B1] Allan CA (2012). OMERO: flexible, model-driven data management for experimental biology. Nat. Methods.

[btu349-B2] Bolte S, Cordelieres FP (2006). A guided tour into subcellular colocalisation analysis in light microscopy. J. Microscopy.

[btu349-B3] Carpenter AE (2012). A call for bioimaging software usability. Nat. Methods.

[btu349-B4] Clynes D (2014). ATRX dysfunction induces replication defects in primary mouse cells. PLoS One.

[btu349-B5] Girish V, Vijayalakshmi A (2004). Affordable image analysis using NIH Image/ImageJ. Indian J. Cancer.

[btu349-B6] Miller CA (2013). Scribl: an HTML5 Canvas-based graphics library for visualising genomic data over the web. Bioinformatics.

[btu349-B7] Sinha AU, Armstrong SA (2012). iCanPlot: visual exploration of high-throughput omics data using interactive Canvas plotting. PloS One.

[btu349-B8] Viangteeravat T (2011). Automated generation of massive image knowledge collections using Microsoft Live Labs Pivot to promote neuroimaging and translational research. J. Clin. Bioinformatics.

